# Evaluation on the application of transcranial Doppler (TCD) and electroencephalography (EEG) in patients with vertebrobasilar insufficiency

**DOI:** 10.1186/s13018-020-01915-z

**Published:** 2020-10-13

**Authors:** Changmin Ke, Chu-na Zheng, Juan Wang, Dongying Yao, Xiaojuan Fang, Yan Luo, Jianglin Wu, Xiaoqing Zheng, Peiping Wang

**Affiliations:** 1 Neurology Department, The Fifth Affiliated Hospital of Sun Yat-sen University, Zhuhai city, 519000 Guangdong Province China; 2 Department of Dermatology, The Fifth Affiliated Hospital of Sun Yat-sen University, No.52, Meihua East Road, Xiangzhou District, Zhuhai City, 519000 Guangdong Province China; 3 Department of Health Management Centre, The Fifth Affiliated Hospital of Sun Yat-sen University, Zhuhai city, 519000 Guangdong Province China; 4 Department of Burns and Plastic Surgery, The Fifth Affiliated Hospital of Sun Yat-sen University, Zhuhai city, 519000 Guangdong Province China; 5 Department of Psychiatry and Psychology, The Fifth Affiliated Hospital of Sun Yat-sen University, Zhuhai city, 519000 Guangdong Province China; 6 Department of Orthopedics, No.6, Dongguan TCM Hospital, Dongguan city, 523000 Guangdong Province China; 7 Department of Urinary Surgery, The Fifth Affiliated Hospital of Sun Yat-sen University, No.52, Meihua East Road, Xiangzhou District, Zhuhai City, 519000 Guangdong Province China

**Keywords:** Vertebrobasilar insufficiency, Clinical diagnosis, Cranial Doppler examination, Electroencephalogram examination

## Abstract

**Background:**

To evaluate the diagnostic value of transcranial Doppler (TCD) and electroencephalography (EEG) in patients with vertebrobasilar insufficiency (VBI) during clinical diagnosis and treatment

**Methods:**

Eighty patients diagnosed with VBI in our hospital from June 2018 to December 2019 were randomly selected as the observation group, and 80 healthy people who received physical examination in the same period were selected as the control group. The abnormal rate, main performance and results, and the peak velocity of blood flow and vertebrobasilar artery blood flow of the two groups were compared.

**Results:**

The abnormal rate of EEG and TCD in VBI patients was 38.75% (31/80) and the 93.75% (75/80), respectively. In TCD examination, ACA, PCA, MCA, and VA of both sides of the observation group were higher than those of the control group, while BA was lower than that of the control group (*P* < 0.05). The Vs, Vd, and Vm on both sides of BA and VA in the observation group were lower than those in the control group, while PI and RI were higher than those in the control group (*P* < 0.05).

**Conclusions:**

TCD examination is highly sensitive to the degree and pattern of cerebral ischemia in VBI patients. EEG examination will define the changes of brain cell function after cerebral ischemia. Therefore, EEG and TCD have their own advantages. The application of TCD and EEG can be considered in the early diagnosis, curative effect, and prognosis evaluation of VBI patients, so as to improve the accuracy of diagnosis and prognosis.

## Introduction

As a common disease, vertebrobasilar insufficiency (vertebro-basal artery insufficiency, VBI) mainly refers to the temporary insufficiency of blood supply or ischemic attack of vertebrobasilar artery, which is usually manifested as recurrent or intermittent attack of nerve dysfunction, making patients have nausea and vomiting, tinnitus, vertigo, and other clinical symptoms [1]. The clinical symptoms and etiology of the disease are complex and diverse, so it is difficult to use conventional diagnosis. TCD is mostly used in recent years. Many studies have confirmed that TCD has significant effect [2], but there are few reports on application of EEG in VBI. In view of this, TCD and EEG will be used to analyze VBI patients in order to evaluate the clinical application value. The aim of our study was to evaluate the diagnostic value of TCD and EEG in patients VBI during clinical diagnosis and treatment.

## Methods

### Patients

Subjects were 80 VBI patients (observation group) and 80 healthy people (control group) who were admitted to our hospital from June 2018 to December 2019. There was no significant difference between the two groups (*P* > 0.05). Comparability is hence established. Refer to Table 1.

**Table 1:** Comparison of general data of the two groups of patients

Group	Cases	Gender [*n* (%)]		Average age (years)	Average body weight (kg)
		Male	Female		
Observation group	80	43 (53.75)	37 (46.25)	44.51 ± 2.03	56.05 ± 6.26
Control group	80	45 (56.25)	35 (43.75)	43.56 ± 2.45	55.06 ± 7.27
*x*^*2*^*/t*	-	0.126		0.158	0.206
*P*	-	0.722		0.874	0.836

### Inclusion and exclusion criteria

VBI: (1) Inclusion criteria: ①the clinical examination (MRI or CT) was in accordance with the diagnosis standard of *vertebrobasilar insufficiency* [3]; ②the reason for disease is clear, such as hypertension, diabetes, and cervical spondylosis; ③patients were examined in 3 days after the onset of the disease; ④consent to participate in the study and family members are informed; ⑤vital signs are stable. (2) Exclusion criteria: ①vertigo caused by ear, eye, and other intracranial diseases; ②with malignant tumor; ③pregnant or lactating women; ④cognitive impairment and mental illness.

Control group: (1) Inclusion criteria: ①being healthy through regular physical examination; ②no history of diabetes, cerebrovascular, and hypertension; ③volunteer for research; ④informed consents were obtained. (2) Exclusion criteria: ①patients with nervous system disease; ②there are vertigo symptoms; ③poor coordination; ④pregnant and lactating women; ⑤data missing and withdrawal.

### TCD and EEG measurements

TCD and EEG were performed in both groups. (1) TCD examination: TCD color transcranial Doppler instrument (produced by Sutter of Germany, model DWL) was used to guide the patients to take their supine position posture and shift into sitting. The professional doctors used 2MH_z_ pulse probe (low frequency), and through the occipital window, the ultra-low arteries (BA, sampling depth of 74–80 mm) and vertebral arteries (VA, sampling depth of 60–70 mm), anterior cerebral arteries (ACA), middle arteries (MCA), and posterior arteries (PCA) examined were used to detect and obtain the blood flow spectrum, and then the peak diastolic velocity (VM), resistance index (RI), systolic velocity (VS), and arterial index (PI) were recorded. (2) EEG examination: the EEG instrument (produced by Shenzhen Delikai, model 10_20 system EEG monitor) was used to place the electrodes according to the international 10/20 system, the time constant was adjusted to 0.3 Hz, the filter wave was adjusted to 35 Hz, the electrodes were placed on the subject’s earlobe, the tracing time was controlled within 30 min, and the conventional monopole and bipolar tracing were performed to guide the α distribution, symmetry, and the frequency, and the ratio of θ to δ of diffuse wave were observed and counted.

### Observation index

The TCD and EEG results of VBI patients were analyzed. The peak velocity of blood flow (ACA, MCA, PCA, RA, and VA) and the blood flow of vertebrobasilar artery (VS, VM, RI, Pi on both sides of BA and VA) were compared with those of the control group. The TCD diagnostic criteria refer to Abreu et al. [4] and other evaluation criteria. The EEG diagnostic criteria refer to Zafonte et al. [5] and other evaluation criteria.

### Statistical analysis

The data was analyzed through the SPSS22.0 software. Measurement data were expressed by ($\overline{\mathrm{x}}$±s), *t* test, count data by (%), chi square test, and *P* < 0.05 means being statistically significant.

## Results

### Comparison of TCD examination between the two groups

**Fig. 1:**
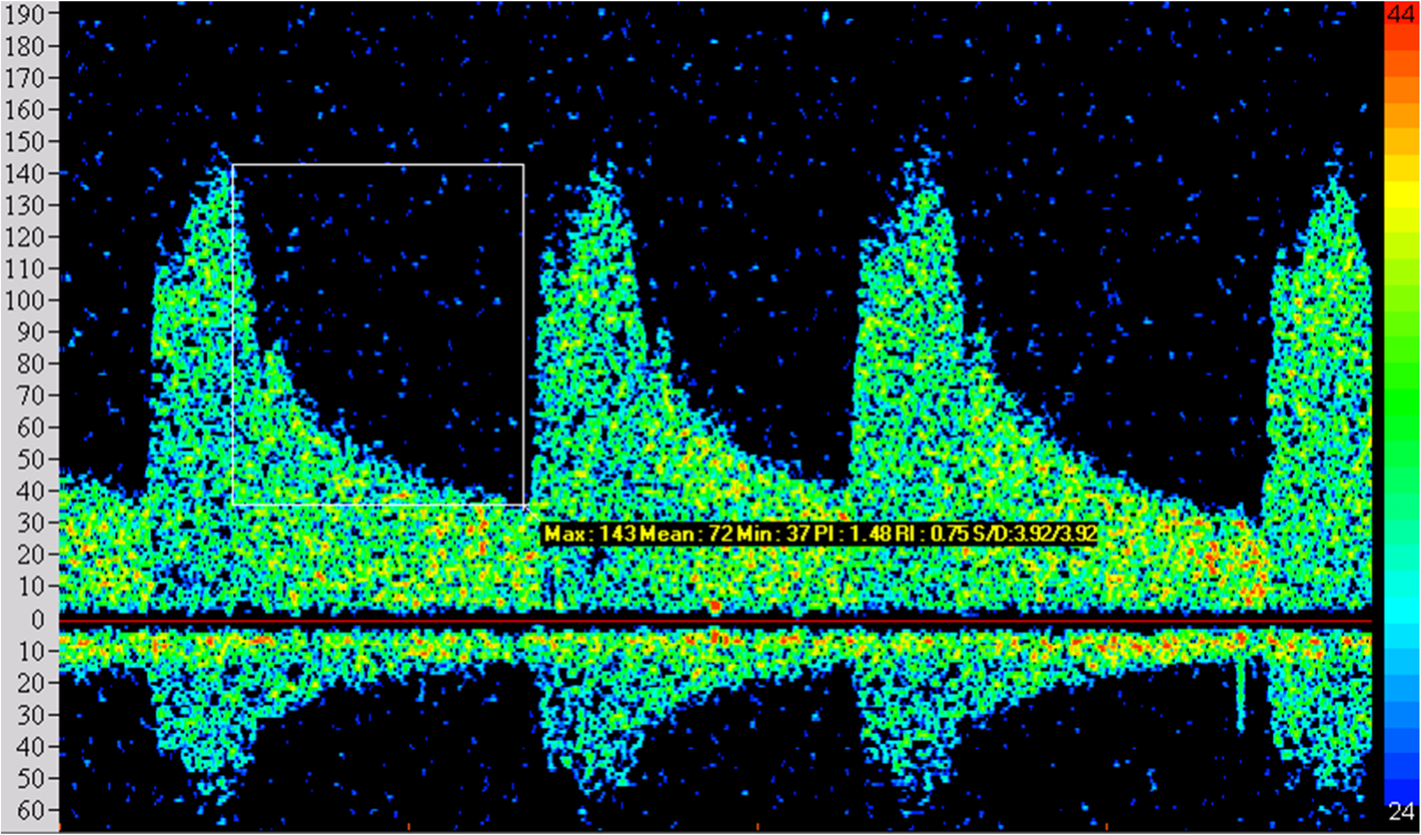
Blood flow spectrum of the control group in TCD examination

**Fig. 2:**
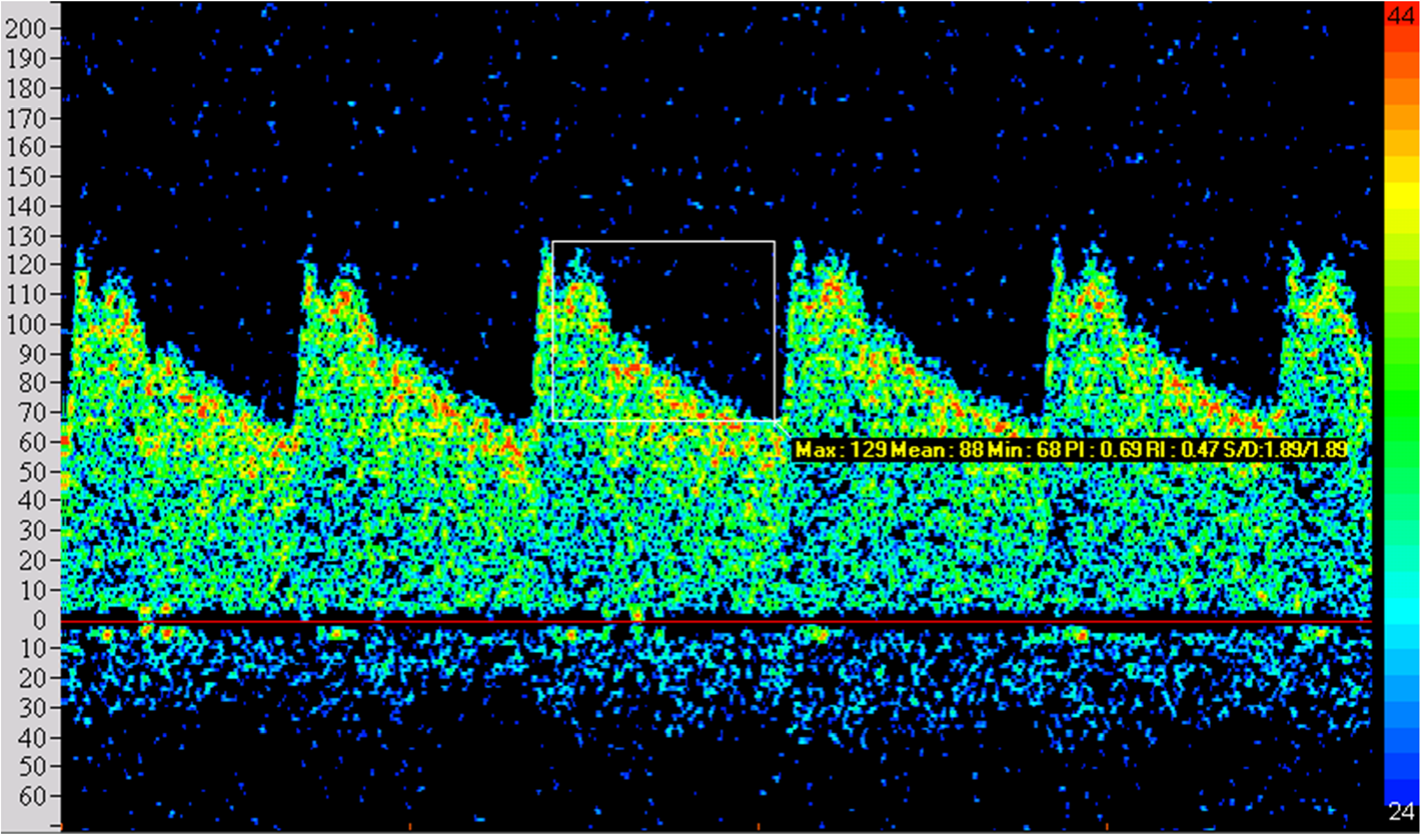
Blood flow spectrum of the observation group in TCD examination

The abnormal rate of TCD in observation group was 93.75% (75/80). The main characteristics were that the blood spectrum was similar to a right triangle, the peak time was delayed and waveform was blunt, the flow velocity on both sides was unbalanced, PI showed a decreasing or increasing trend, the abnormal frequency spectrum S1 ≤ S2, and the image comparison between observation group and control group is shown in Figs. 1 and 2. The peak velocity of MCA, ACA, PCA, and VA in the left and right sides of the observation group were higher than those in the control group, while BA was lower than that in the control group, with a statistically significant difference (*P* < 0.05). Refer to Table 2.

**Table 2:** Comparison of peak velocity of blood flow between two groups in TCD examination ($\overline{\mathrm{x}}$ ± s, cm/s)

Group	Side	ACA	MCA	PCA	BA	VA
Observation group (*n* = 80)	Left	60.52 ± 9.45^*a*^	78.64 ± 10.46^*a*^	48.78 ± 8.42^*a*^	44.52 ± 5.56^*a*^	33.64 ± 10.12^*a*^
	Right	60.56 ± 10.24^*a*^	80.56 ± 9.14^*a*^	48.36 ± 9.12^*a*^		33.95 ± 11.12^*a*^
Control group (*n* = 80)	Left	74.13 ± 9.46	89.00 ± 10.12	57.23 ± 6.03	53.12 ± 8.46	45.13 ± 9.36
	Right	75.00 ± 9.12	89.45 ± 10.72	57.16 ± 6.17		43.25 ± 9.69

Notes: compared with the control group, ^*a*^*P*<0.05

### Comparison of blood flow of vertebrobasilar artery between two groups under TCD examination

The Vs, Vd, and Vm of BA and VA in the observation group were lower than those in the control group, while PI and RI were higher than those in the control group (*P* < 0.05). Refer to Table 3.

**Table 3:** Comparison of blood flow change of vertebrobasilar artery between two groups in TCD examination ($\overline{\mathrm{x}}$ ± s, v/cms^−1^)

Group	Artery	Side	Vs	Vd	Vm	PI	RI
Observation group (*n* = 80)	BA		44.52 ± 5.53^*a*^	24.38 ± 8.39^*a*^	32.46 ± 9.46^*a*^	0.73 ± 0.12^*a*^	0.67 ± 0.12^*a*^
	VA	Left	33.63 ± 10.09^*a*^	20.30 ± 6.69^*a*^	24.36 ± 7.38^*a*^	0.79 ± 0.12^*a*^	0.65 ± 0.07^*a*^
		Right	33.95 ± 11.02^*a*^	18.06 ± 6.45^*a*^	25.18 ± 8.36^*a*^	0.77 ± 0.13^*a*^	0.66 ± 0.16^*a*^
Control group (*n* = 80)	BA		53.92 ± 9.86	27.56 ± 8.95	36.46 ± 8.46	0.70 ± 0.08	0.62 ± 0.05
	VA	Left	45.36 ± 9.34	20.33 ± 7.63	30.14 ± 7.46	0.71 ± 0.09	0.58 ± 0.06
		Right	43.26 ± 9.78	22.36 ± 7.56	28.60 ± 7.08	0.71 ± 0.11	0.56 ± 0.03

Notes: compared with the control group, ^*a*^*P*<0.05

### Analysis on EEG examination of the two groups

The abnormal rate of EEG in the observation group was 38.75% (31/80), including 16 cases of edge state EEG, accounting for 51.61% (16/31); 10 cases of mild abnormal EEG, accounting for 32.26% (10/31); and 5 cases of moderate abnormal EEG, accounting for 16.13% (5/31). The changes of EEG in limbic state showed a decrease of α activity, lack of α wave in the occipital and parietal regions, and poor regulation and amplitude modulation. Among them, there were 7 cases of α wave generalization or migration with α frequency between 8 and 10 Hz. The mild EEG changes showed that the background EEG lacks α activity, and the short-term or paroxysmal group produced 30–50 μV, 5–8 c/s θ activity. The moderate abnormal EEG showed that there were 40~70 μV, θ rhythm, and scattered δ wave in the background EEG. In eye open-and-close test, there were 5 patients (16.13%) with incomplete inhibition of α wave. In hyperventilation, there was no significant change in the background EEG after hyperventilation, and there was a slight diffusion increase in the θ wave and δ wave of slightly abnormal and moderately abnormal EEG. In the control group, EEG showed marginal state in 6 cases, accounting for 7.50% (6/80); slight abnormal EEG in 1 case, accounting for 1.25% (1/80); and no other abnormal special changes were found.

## Discussion

As a common type of intracranial vascular disease, VBI mainly includes reversible ischemic neuropathy and transient ischemic attack neuropathy. According to the anatomical standard, the vertebrobasilar artery system is composed of vertebral arteries on the left and right sides and inferior pontine basilar arteries. It is mainly responsible for supplying blood to the brainstem, cerebellum, and occipital part of the brain [6]. When VBI occurs, the blood flow in the blood supply area decreases, resulting in insufficient blood supply, metabolic damage of neuron cells, and weakening of synaptic function. If the intervention is not carried out in time to make the recurrent attack, this change will persist for a long time. Even if it does not recover in the intermittent period, it is easy to promote the occurrence of vertebral basilar artery ischemic change. Brunser et al. [7] reported that there were many branches in the vertebrobasilar artery, which would connect the middle cerebral artery and the anterior cerebral artery through the Willis ring. When VBI has some clinical symptoms, such as vertigo, the hemodynamics in the brain will change, the cerebral blood flow will be reduced, and the brain function will be correspondingly impaired.

At present, a large number of studies have confirmed the pathogenesis of VBI, mainly including the following points: ①cervical degenerative changes, cervical spondylosis, neck inflammation and neck muscle brain damage, etc., which will lead to compression of the vertebral artery. When the position of the head changes, it will stimulate the sympathetic nerve of the neck, and then induce the spasm of the vertebral artery, resulting in change of blood flow velocity of artery; ②due to the influence of their diseases, such as hyperlipidemia, hypertension, and diabetes, atherosclerosis will occur, involving all blood vessels and their branches, leading to stage vascular stenosis, resulting in corresponding changes in blood flow speed and spectrum morphology [8, 9]. Previously, there was no simple and effective method for the detection of VBI, but in recent years, with the development and progress of medical technology and the emergence of TCD, it provides an accurate, noninvasive, and simple method for the diagnosis of VBI. Through the diagnosis results, it will be clear about the blood flow status of single vessel, such as diastolic speed, boost index, contraction speed, and vascular compliance, which is helpful for accurate reflection of the subtle changes of VBI [10, 11]. In TCD observation index, Vd and Vm are mainly used to reflect the hemodynamic changes of the measured blood vessels, PI, RI, and pulse parameters, and to evaluate the arterial toughness and compliance. In reflecting the cerebral vascular pulsation, the boost index is more sensitive to vascular compliance than the pressure index, which fully reflects the changes of cerebral vascular resistance. The study showed that the abnormal rate of TCD in the observation group reached 93.75%, which was similar to that of 89.46% of VBI patients in the research results of Wu et al. [12], and demonstrated that TCD had a high sensitivity in the diagnosis of VBI and would reflect the abnormal situation of vertebrobasilar artery. In TCD examination, VM serves a high-value index. When the flow rate is too slow, it means that the blood supply of blood vessels is insufficient, and when the flow rate is faster, it means that the blood vessels are narrow or spasm. Both of them will lead to the decrease of cerebral circulation blood flow, which is also verified in the study results that Vs, Vd, and Vm in the observation group are lower than those in the control group.

EEG is mainly used to reflect the functional state of brain cells. It was found in this study that the abnormal rate of EEG in VBI patients was 38.75%, which was manifested in decrease of α index. There was no advantage in occipital region. The existence of α wave was moving forward. The regulation of α wave amplitude was poor, among which the increase of θ wave was 32.26% and 16.13% in mild and moderate abnormality. There was no other serious abnormality EEG and other characteristic disease wave, which showed that the characteristic of EEG was poor in VBI diagnosis. However, considering that brain cells are particularly sensitive to hypoxia and ischemia, when the intracranial artery is in a state of insufficient blood supply for a long time and there is arteriosclerosis, it is easy to cause hypoxia and hypoxia of brain cells, resulting in functional damage of brain cells and changes in electrophysiological activities. Previous research [13] showed that the vertebrobasilar system would supply blood to the cerebral cortex to keep electrophysiological activity of the blood supply site at a normal level. Under normal circumstances, EEG shows that α rhythm is at a superior point occipital region, with good amplitude regulation and good synchronous symmetry on both sides. VBI showed abnormal changes in α wave, which also reflected that ischemic changes may occur in the cortex of blood supply to some extent [14, 15]. The study shows that TCD has a higher abnormal rate than EEG, which indicates that TCD is highly sensitive in the abnormal changes of cerebral blood volume. It will not only identifies the changes of cerebral blood flow in the early stage, but also shows the degree and scope of cerebral ischemia. TCD can be used to detect the disease when it occurs in cerebral blood flow. Zamani et al. [16] showed that TCD was better than EEG in identifying the diseased vessels, ischemic degree, and anatomical location. However, EEG may find the changes of brain wave in VBI patients from the perspective of electrophysiology. It can evaluate the functional state of brain cells according to the increase of slow waves such as α wave, δ wave, and θ wave, reflecting the situation of cerebral cortex ischemia. In contrast, TCD examination is mainly to provide objective reference basis for VBI patients, such as the degree and scope of cerebral ischemia. EEG examination is more convenient and will find the changes of cell function upon cerebral ischemia. Although the positive rate of VBI patients in this study is significantly different in the two examination methods, they reflect the pathological and physiological status of VBI from different directions, which means that the two examination methods are complementary to some extent.

There were also some limitations in this study. First, the sample size in this study was relatively small. Further study with lager sample size was needed. Second, the combination application of EEG and TCD has not been evaluated.

## Conclusion

In conclusion, TCD examination is highly sensitive to the degree and pattern of cerebral ischemia in VBI patients. EEG examination will define the changes of brain cell function after cerebral ischemia. Therefore, EEG and TCD has its own advantages. The application of TCD and EEG can be considered in the early diagnosis, curative effect, and prognosis evaluation of VBI patients, so as to improve the accuracy of diagnosis and prognosis.

## Data Availability

Not applicable.

## Abbreviations

TCD: Transcranial Doppler
EEG: Electroencephalography
VBI: Vertebrobasilar insufficiency
